# The Effects of Colicin Production Rates on Allelopathic Interactions in *Escherichia coli* Populations

**DOI:** 10.3390/microorganisms7110564

**Published:** 2019-11-14

**Authors:** Lusine Ghazaryan, Itamar Giladi, Osnat Gillor

**Affiliations:** 1 Zuckerberg Institute for Water Research, Jacob Blaustein Institutes for Desert Research, Ben-Gurion University of the Negev, Sede Boqer Campus, 84990 Midreshet Ben-Gurion, Israel; lusin@post.bgu.ac.il; 2 Mitrani Department of Desert Ecology, Swiss Institute for Dryland Environmental and Energy Research, Jacob Blaustein Institutes for Desert Research, Ben-Gurion University of the Negev, Sede Boqer Campus, 84990 Midreshet Ben-Gurion, Israel; itushgi@bgu.ac.il

**Keywords:** competition, bacteriocin, colicin, rate dependent, *Escherichia coli*, GFP, planktonic

## Abstract

Allelopathic interactions mediated by bacteriocins production serve microorganisms in the never-ending battle for resources and living space. Competition between the bacteriocin producer and sensitive populations results in the exclusion of one or the other depending on their initial frequencies, the structure of their habitat, their community density and their nutrient availability. These interactions were extensively studied in bacteriocins produced by *Escherichia coli*, the colicins. In spatially structured environments where interactions are local, colicin production has been shown to be advantageous to the producer population, allowing them to compete even when initially rare. Yet, in a well-mixed, unstructured environment where interactions are global, rare producer populations cannot invade a common sensitive population. Here we are showing, through an experimental model, that colicin-producers can outcompete sensitive and producer populations when the colicin production rates are enhanced. In fact, colicin production rates were proportional to the producer competitive fitness and their overall success in out-competing opponents when invading at very low initial frequencies. This ability of rare populations to invade established communities maintains diversity and allows the dispersal of beneficial traits.

## 1. Introduction

Microbial communities’ continuous competition for shared limited resources and space is often resolved by the production of toxins [1]. Coexistence of toxin-producer and susceptible, non-producer populations has been studied in many bacteria and yeast communities [2]. Competition between toxin producer and sensitive populations results in the exclusion of one or the other, depending on their initial frequencies [3,4], the structure of their habitat [5], the community density [6] and nutrient availability [7].

One major group of toxins that is directly associated with bacterial allelopathic interactions are the bacteriocins, narrow spectrum proteinaceous toxins that kill only closely related strains that compete over the same resources and space with the bacteriocin-producing cells [2]. In spatially structured environments, where cells are sessile and interactions are local, bacteriocin-producing colonies kill neighboring sensitive cells and are rewarded by an increase of available resources in their vicinity [5]. Therefore, producers can out-compete sensitive populations even when initially rare [8,9]. However, the producer competitive advantage is reduced either at low densities, probably because of reduced interactions [6], or when populations are clonally segregated, and thus susceptible species can coexist or even outcompete producers [10]. Besides, killing is costly and a lack of competition with sensitive cells reduces the fitness of the producers to a point where they are out-competed by non-producer, sensitive cells [5].

The killing of sensitive cells in a well-mixed environment where cells are randomly distributed and interactions are global would result in equal distribution of the released nutrients, profiting the entire community [1]. There is no direct benefit to the producers’ population, yet they carry the metabolic cost of bacteriocin production, impairing their fitness, and therefore, the producers will out-compete sensitive population only when their initial frequency exceeds a certain threshold [8]. Yet, even if the producers start at high initial frequencies, they will be outcompeted by sensitive cells when the density of the community is low, because the producers could not be spatially confined to compensate for the cost of production [6,11]. An additional factor that may affect producer-sensitive interactions is the availability of nutrients [12]. In nutrient rich environments, producers will out-compete sensitive cells, but low nutrient environments are advantageous to sensitive cells, and they prevail [1,7]. A twist in these interactions are competing bacteriocin producers that were shown to be simultaneously lethal and sensitive to each other [13,14]. The allelopathic interactions, mediated by these bacteriocins play both defensive and offensive roles. On the one hand, they prevent the invasion of populations into established producer populations, and on the other, they propel the colicins producers’ advancement to out-compete nearby sensitive populations and in the process liberate space and resources [9,15,16].

In a previous study we explored allelopathic interaction using colicins, bacteriocins produced by *Escherichia coli*. We have shown that a competition between two different colicin hosts, one weaker than the other, results in the weaker beating the more potent colicin [14]. Such interaction was termed ‘survival of the weakest’, arguing that in an environment where species A is cohabiting with species B and C that are stronger (even slightly), then A has the highest probability of surviving [17,18]. This outcome, demonstrated in both in vivo and in silico models, might be counterintuitive, because although A is considered the “weakest” species, it is robust, especially in large, clonally segregated populations [18]. We speculated that colicins play a dual role in competition, they are both toxic to their competitors but also regulate their opponents’ colicin production [14]. However, the ‘survival of the weakest’ hypothesis raises the question: how do the ‘strong’ competitors persist in a given community if they are regularly outcompeted by their weaker opponents? Killing of sensitive populations is a function of the frequency and cost to the producers [19], the spatial structure of the ecosystem [5], the effect of the colicin on target cell growth [20] and the relative importance of allelopathy on resource competition [12]. However, we propose that another important component was overlooked, the colicin production rates that could transform the outcome of the allelopathic interactions.

In this study we used colicins that kill competing strains through one of a variety of mechanisms, i.e., by nucleic acid degradation [21]. In a previous study, we established that the production rates of these colicins vary among producers [22]. Here, we used an experimental model to demonstrate that in a well-mixed environment, changing colicin production rates are tightly correlated with the initial frequency required to outcompete opposing populations, may they be colicin -sensitive or -producer. This allows producers to invade new environments and challenge established residents, even when they are initially very rare.

## 2. Materials and Methods

### 2.1. Bacterial Strains, Plasmids and Growth Conditions

The bacterial strains and plasmids used in this study are listed in Table 1. Bacteria were grown in minimal (M9; Sigma, St Louis, MO, USA) and rich (Luria Bertani (LB); Difco, Lawrence, KS, USA) media at 37 °C in a shaking incubator (New Brunswick Instruments; Edison, NJ, USA) at 250 rpm. When relevant, the media were supplemented with ampicillin and kanamycin (Sigma) at final concentrations of 100 and 30 µg mL^−1^, respectively.

### 2.2. General DNA Techniques

Plasmid DNA was isolated with a plasmid extraction kit (Bioneer, Seoul, Korea) and transformed to *E. coli* strain BZB1011 [23], thus providing an isogenic host to the different bacteriocin encoding plasmids. Transformants were selected according to bacteriocin production, and their identities were confirmed by PCR and sequencing analyses.

### 2.3. Bacteriocin Production Assay

Bacteriocin assays were performed as previously described [25,26] with slight modifications. Briefly, the bacteriocinogenic strains (Table 1) were cultivated overnight in an M9 medium, diluted 1:100, re-inoculated to a fresh medium and incubated till reaching the early-exponential phase (Absorbance 600 of ~0.07; absorbance measured using Infinite 200M Tecan, Männedorf, Switzerland). The cultures were then supplemented with 50 ng mL^−1^ Mitomycin C (MitC; Sigma), a DNA damaging agent. After 5 h of incubation, the cells were harvested and treated with 6.7% chloroform (Sigma), vortexed for 1 min, and the cell debris were removed by centrifugation (Eppendorf 5415D, Hamburg, German) for 3 min at 16,000× *g*. The supernatants were used for bacteriocin assays. The crude colicin extracts were two-fold diluted, and 10 µL aliquots were placed on LB plates overlaid with the colicin-sensitive *E. coli* strain BZB1011, which is susceptible to all the colicins used in this study. The plates were incubated for 12 h in an incubator (Tuttnauer, Breda, The Netherlands) at 37 °C. Bacteriocin production rates were visualized as a clearing zone within the bacterial lawn.

### 2.4. Competition Assays

The strains used for the expression-based assays are listed in Table 1. The strains harboring pColE2, pColE8 and pColE7 plasmids (ID: E2, E7 and E8, respectively) were competed against a colicin-sensitive strain carrying the reporter vector *pUArrnB* [24] (ID: WT-GFP) and against a colicin A producing strain together with *pUArrnB* [14] (ID: A-GFP). The strains WT-GFP and A-GFP were incubated overnight in an M9 medium at 37 °C in a shaking incubator (New Brunswick Instruments) at 250 rpm. In the morning, the strains were re-inoculated in a fresh medium at 1:100 dilution, and cultured to the exponential phase (OD600 of ~0.07; Infinite 200M). Subsequently, a fresh culture of the strains was distributed into semi-transparent 96-well microplates (Greiner, Frickenhausen, Germany) and incubated together with strains E2, E7, E8 and the plasmid-free *E. coli* strain BZB1011 (ID: WT), at starting frequencies of 50%, 10%, 1%, 0.1%, 0.01%, 0.001% and 0.0001%. As a control, WT-GFP and A-GFP were propagated alone at the same conditions and volume. The plates were covered with sealing tapes (Thermo Fisher, Waltham, MA, USA), incubated at 37 °C in a plate reader (Infinite 200M) for fluorescence and absorbance measurements, and monitored for 20 h at 15-min intervals. Increase in fluorescence indicated that the reporter was either outcompeted or co-existed with its putative competitor. We note that the reporter gene used, *gfpmut2* [24], is un-degradable such that cell death would not diminish fluorescence levels [27]. Therefore, a halt in fluorescence increase translates to cessation in WT-GFP and A-GFP growth. For each strain, the maximum fluorescence emission was plotted over time, as the mean of three biological replicate assays were each performed in duplicate. In addition, the survival rate of the common competitors, WT-GFP and A-GFP, at each pairwise competition and their related frequency were calculated as the percentage of emitted fluorescence from the maximum fluorescence by the lone reporter strains. In addition, for validation at the end of each experiment, cells were enumerated by inoculation [28]. The survival rate was calculated at each monitored time point. All experiments were repeated independently at least four times in duplicates. Statistical analyses were performed with Statistica (v. 13.5.0, StatSoft, Hamburg, Germany) using general linear models with concentration and strain as fixed factors and the experiment as a random block.

### 2.5. Relative Fitness of the Colicinogenic Strains

To measure the relative fitness (*w*), we competed the colicinogenic strains and their plasmid free ancestral strain (BZB1011) against an isogenic strain harboring the reporter plasmid pUA*rrnB* (Table 1) at a 1:100 ratio in an M9 medium. We quantified the abundance of the strains at the co-inoculation time and 24 h later at the end of the competition experiment. The fitness of the colicinogenic strains relative to that of the reporter strain was calculated as previously described [29]. All frequencies were replicated six times.

## 3. Results

**Figure 1:**
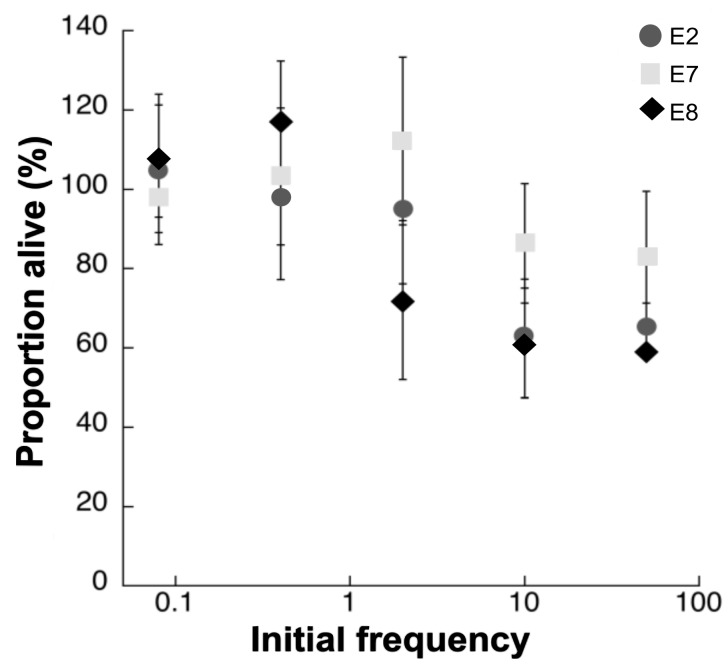
Survival of WT-GFP (Table 1) following four hours of incubation with colicin-producing populations. The rate of survival, reported as proportion of live cells, is indicated as the percentage of fluorescence emitted by the reporter strain incubated at different initial frequencies with the populations producing colicins E2 (circle), E7 (square) and E8 (diamond) divided by the maximum fluorescence emitted by WT-GFP propagated alone. Each point is the mean of at least three independent experiments performed in duplicates.

The mode of action of all four colicins used in this study is similar; they all kill their competitors by DNA degradation [22,30], yet, they vary in their colicin-expression rate (Table 2). The basal level of colicin expression differs when cell populations are not induced (Table 2). In fact, the basal expression rates of the populations carrying colicins E2 and E8 is four- and six-fold higher, respectively, than the expression rate of the population harboring colicin E7 (Table 2). We used these populations that naturally vary in their colicin expression and competed them and the isogenic, colicin-sensitive *E. coli* host strain BZB1011 against the colicin-sensitive reporter strain *pUArrnB* (Figure 1) and against the colicin A producing strain tagged with the reporter plasmid pUA*rrnB*.

**Figure 2:**
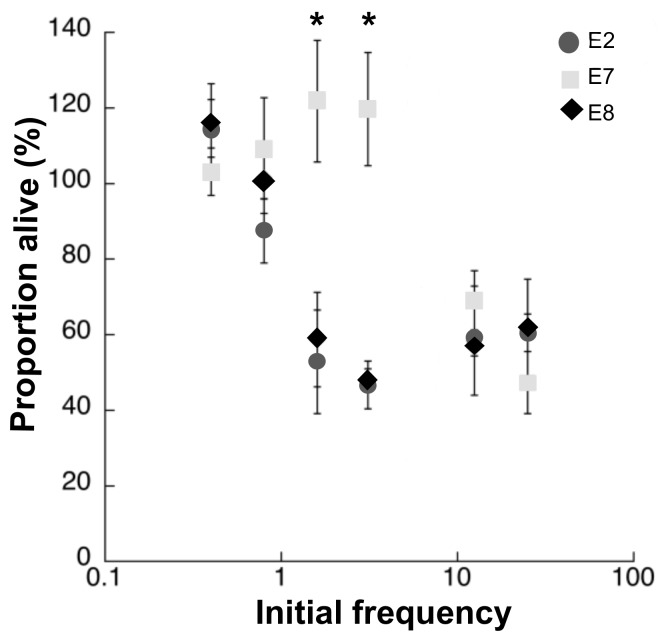
Survival of A-GFP (Table 1) following four hours of incubation with colicin-producing populations. The rate of survival, reported as proportion of live cells, is indicated as the percentage of fluorescence emitted by the reporter strain incubated at different initial frequencies with the populations producing colicins E2 (circle), E7 (square) and E8 (diamond) divided by the maximum fluorescence emitted by A-GFP propagated alone. Asterixis mark statistically significant (* *p* < 0.05) difference between the tested colicins. Each point is the mean of three independent experiments performed in duplicates.

We first monitored in real time the survival of the susceptible, fluorescent, non-producing population (reported as proportion alive) incubated in the presence of the colicinogenic strains and the plasmid-free, isogenic, host strain at different initial concentrations (Figure 1). We competed weaker against stronger colicinogenic strains and have detected that stronger strains inhibit competition by quickly killing their opponents, even when they are initially rare (Figure 2). The survival of both reporter strains (WT-GFP and A-GFP) was significantly lower when incubated with the ‘high’ expresser strains, E2 and E8, than with the ‘low’ expresser strain, E7 (*p* < 0.01) (Figure 1 and Figure 2). We note that when the WT-GFP was competed against the WT, no decrease in its fluorescence emissions was detected. Accordingly, there was a significant difference between the survival rate of the WT-GFP incubated with the colicinogenic strains and the susceptible strain (*p* < 0.01). When competed against WT-GFP, the low expresser, E7, was shown to successfully compete only when its initial frequency was above 2% (Figure 1), while the stronger competitor, E8, inhibited WT-GFP at initial frequencies as low as 0.5%. E2 was shown to successfully compete in between the initial concentrations of E7 and E8 (Figure 1). However, competition against A-GFP showed that the low expresser, E7, can successfully compete at higher initial frequencies above 10% (Figure 2). Likewise, the stronger competitors, E2 and E8, inhibited A-GFP at initial frequencies above 1%. Accordingly, there was a significant difference between the survival rate of the A-GFP incubated with the colicinogenic strains and the susceptible strain (*p* < 0.01).

**Figure 3:**
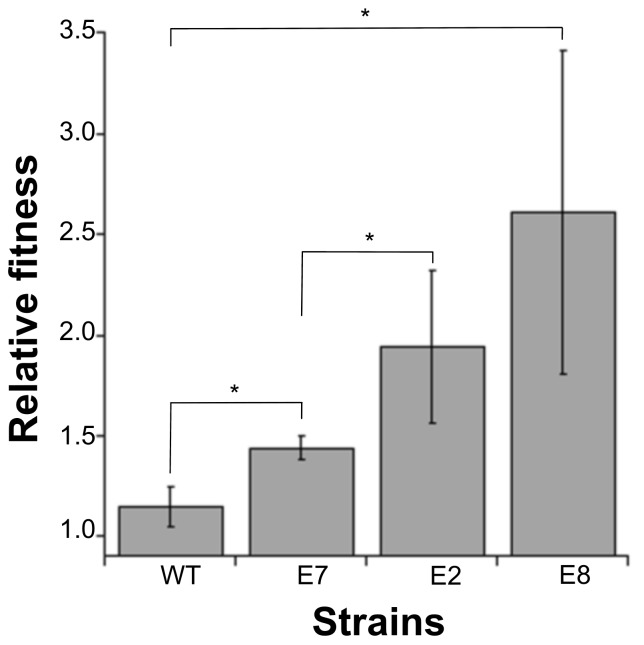
Colicin-producing strains and their susceptible host strain were mixed at approximately a 1:100 ratio with WT-GFP and then cultured to stationary phase. Confirmed initial and final abundance were used to calculate the relative fitness of each strain compared to the reporter strain. Asterixis mark statistically significant (* *p* < 0.05) difference between the tested colicins. The means and standard deviation of six independent experiments are shown.

To identify the cost of colicin production, we compared the growth rate of the strains used in this study (Table 3) and found differences in the fitness that could be related to the expression rate (*p* < 0.05). Yet, when we competed the colicin-producing strains and the susceptible strain with the reporter strain at a ratio of 1:100, we found that high expression carries fitness advantage. The high colicin producers showed advantage against the susceptible strain under these conditions (Figure 3) that were proportional to the colicin expression rate (Table 3), such that high colicin expression (E8) confers the largest fitness advantage (*w* = 2.61 ± 0.80, *n* = 6) while lower expression (E2) confers lower advantage (*w* = 1.94 ± 0.38, *n* = 6), and the lowest expression rate (ColE7) confers the lowest fitness advantage (*w* = 1.44 ± 0.06, *n* = 6). These results confirm that colicin expression involves growth rate cost but can confer fitness advantage when in direct competition with a susceptible or colicinogenic strain.

## 4. Discussion

We investigated expression dependency in toxin-mediated competitive interactions using colicins as our model system. The relative fitness of colicin producers involved in direct competition with susceptible isogenic strains was shown to vary according to the initial frequencies of the producers [11]. It was shown that fitness is reduced at low and high starting frequencies because increase or decrease in the competitor’s frequency may be outweighed by the cost of production, thus suggesting that producers have a fitness advantage at intermediate spatial relatedness in both structured and unstructured environments [4,31,32]. Yet, these experimental and related theoretical models [13,31] assumed uniformity in the production rate. We confirmed our prediction that differential killing is directly linked with the initial frequencies, yet they present a scenario in which the outcome of colicin-mediated dual competition is linked not only to the initial frequency, spatial structure and nutrient availability, but also to the indigenous production rate (Table 2). We have shown that competitive fitness increases with production rate (Figure 3) such that high producers will outcompete their opponents, even when initially rare (Figure 2 and Figure 3).

We demonstrated that in well-mixed environments, producers could invade both susceptible and colicinogenic population at lower initial frequencies than previously reported [8,33] if their colicin expression rate is high enough (Figure 1 and Figure 2). Previous studies, both experimental and theoretical, assumed colicin production rates to be constant and explored the changing initial frequencies [8,31], nutrient availability [1,7], the spatial structure [5] and community density [6] to determined their role in competitive interactions. We tested isogenic strains that differed only in their colicin types. The colicins tested were shown to differ in their production rates (Table 2), and we were able to assess under controlled conditions colicin’s role in banning competitors (Figure 1 and Figure 2) and increasing the host’s competitive fitness (Figure 3).

One could assume that high production rates are advantageous, especially upon invasion into an established population in an attempt to dominate its resources. In this scenario, the invader population that produces colicin at high rates would have the upper hand, outcompeting their adversaries even when initially rare. Yet, studies have shown that weaker strains could outcompete stronger strains when competed in large segregated populations [17,18]. We propose that the survival of the weak depends on how fierce its stronger opponent is, which would determine whether the weak would become extinct, and thus unable to compete. Here, we have shown that very strong competitors would quickly kill their adversaries, driving them to extinction (Figure 1 and Figure 2).

We have shown that high producers defeat their opponents, yet how do they establish and persist in their new niche? Upon colonizing and replacing their opponent, the elevated production rates could prove a liability and lower the conqueror’s fitness compared to adjacent, non-producing populations. Future studies would prove whether elevating colicin production rates is a sustainable strategy for producers both when conquering a new niche and when establishing or defending it. One clue could be the difference between bacteriocin expression levels in planktonic and biofilm cultures [34]. As cells were establish in their new environment and formed a biofilm, a very different gene expression profile was reported compared to planktonic cells [35,36]. These changes could reduce toxin production, lowering metabolic costs and elevating the fitness of the sessile population, thus maintaining diversity in sessile communities, and it allows for the dispersal of beneficial traits. As colicins are effective inhibitors to enteric pathogens [37,38], our findings could also be used to develop means for the successful and sustainable inhibition of pathogens.

## Figures and Tables

**Table 1:** *E. coli* strain and plasmids used in the presented study. All mentioned plasmids were applied in *E. coli* strain BZB1011.

Bacterial Strains/ Plasmids		Relevant Properties	Ref.
**Bacterial Strains**	**ID**		
BZB1011	WT	F-, λ-, gyrA586(Nal^R^), in(*rrnD*-*rrnE*)1, rpsL-(str^R^), rph^−1^	[23]
**Plasmids**			
pUA*rrnB*	WT-GFP	pUA*rrnB*::gfp, Kn^r^	[24]
ColA-pUA*rrnB*	A-GFP	*caa*, *cai*, *cal* (colicin A) & pUA*rrnB*	[14]
PColE2 (pColE2-P9)	E2	*ce2a*, *ce2i*, *ce2l* (colicin E2)	[23]
PColE7 (pColE7-K317)	E7	*ce7a*, *ce7i*, *ce7l* (colicin E7)	[23]
PColE8 (pColE8-J)	E8	*ce8a*, *ce8i*, *ce8l* (colicin E8)	[23]

Abbreviations sorted alphabetically: GFP—green fluorescent protein; ID—identification; Ref.—references; WT—wildtype.

**Table 2:** Percentage of colicin producing cells within a colicinogenic *E. coli* population.

Colicin Type	Rate of Colicin Expressing Cells (%)	
	Late log	Stationary
A	2.7 ± 1.2	3.7 ± 1.5
E2	18.3 ± 6.8	21.1 ± 8.5
E7	3.4 ± 1.4	5.7 ± 0.5
E8	14.6 ± 2.0	28.1 ± 6.5

Colicin producing cells were marked with fluorescence, grown in minimal medium and numerated within a given population at the late log and stationary phases. Each measure represents the mean ± standard deviation of at least four biological replicates.

**Table 3:** Growth rate of *E. coli* strains incubated in minimal medium at 37 °C.

*E. coli* Strains			Growth Rate
Strain	Plasmid	ID	
BZB1011	ColE2	E2	0.34 ± 0.02
	ColE7	E7	0.34 ± 0.03
	ColE8	E8	0.38 ± 0.07
	pUA*rrnB*	WT-GFP	0.41 ± 0.07
	ColA-pUA*rrnB*	A-GFP	0.35 ± 0.03
		WT	0.41 ± 0.06

Each measure represents a mean ± standard deviation (rounded to two decimal points) of at least three biological replicates. Growth rate is expressed in generations per hour.
